# The genome sequence of a cased caddisfly, *Mystacides longicornis *(Linnaeus, 1758)

**DOI:** 10.12688/wellcomeopenres.22272.1

**Published:** 2024-06-13

**Authors:** Derek Coleman, Natural History Museum Genome Acquisition Lab, Darwin Tree of Life Barcoding collective, Wellcome Sanger Institute Tree of Life Management, Samples and Laboratory team, Wellcome Sanger Institute Scientific Operations: Sequencing Operations, Wellcome Sanger Institute Tree of Life Core Informatics team, Tree of Life Core Informatics collective, Darwin Tree of Life Consortium

**Affiliations:** 1 Independent researcher, Carshalton, England, UK

**Keywords:** Mystacides longicornis, cased caddisfly, genome sequence, chromosomal, Trichoptera

## Abstract

We present a genome assembly from an individual male *Mystacides longicornis* (cased caddisfly; Arthropoda; Insecta; Trichoptera; Leptoceridae). The genome sequence is 665.1 megabases in span. Most of the assembly is scaffolded into 20 chromosomal pseudomolecules, including the Z sex chromosome. The mitochondrial genome has also been assembled and is 15.75 kilobases in length.

## Species taxonomy

Eukaryota; Opisthokonta; Metazoa; Eumetazoa; Bilateria; Protostomia; Ecdysozoa; Panarthropoda; Arthropoda; Mandibulata; Pancrustacea; Hexapoda; Insecta; Dicondylia; Pterygota; Neoptera; Endopterygota; Amphiesmenoptera; Trichoptera; Integripalpia; Brevitentoria; Leptoceroidea; Leptoceridae; Leptocerinae; Mystacidini; *Mystacides*; *Mystacides longicornis* (Linnaeus, 1758) (NCBI:txid699848).

## Background

*Mystacides longicornis* is a cased caddis in the family Leptoceridae, which are easily recognised by their very long antennae. The commonest form of the adult is also easily recognised, having wings with dark bands on a fawn background; the other form has plain brown wings. Other features making identification easy are the red eyes and black hairy maxillary palps that protrude outwards ( Barnard & Ross, 2012). The larval case is straight or slightly curved, composed of sand grains and plant fragments. Often fragments of the case project beyond the case, presumably to make them less easy for predators to swallow. The larva is omnivorous and can be found in both still and flowing water with the adults flying from May to September ( Wallace *et al.*, 2003). It is widespread and very common In England and Wales but less so in Scotland ( NBN Atlas Partnership, 2024). It is widespread in Europe ( O’Connor, 2015).

The genome of *Mystacides longicornis* was sequenced as part of the Darwin Tree of Life Project, a collaborative effort to sequence all named eukaryotic species in the Atlantic Archipelago of Britain and Ireland. Here we present a chromosomal-level whole genome sequence for *Mystacides longicornis*, based on one male specimen from Lea Broad, England, UK.

## Genome sequence report

**Figure 1.**
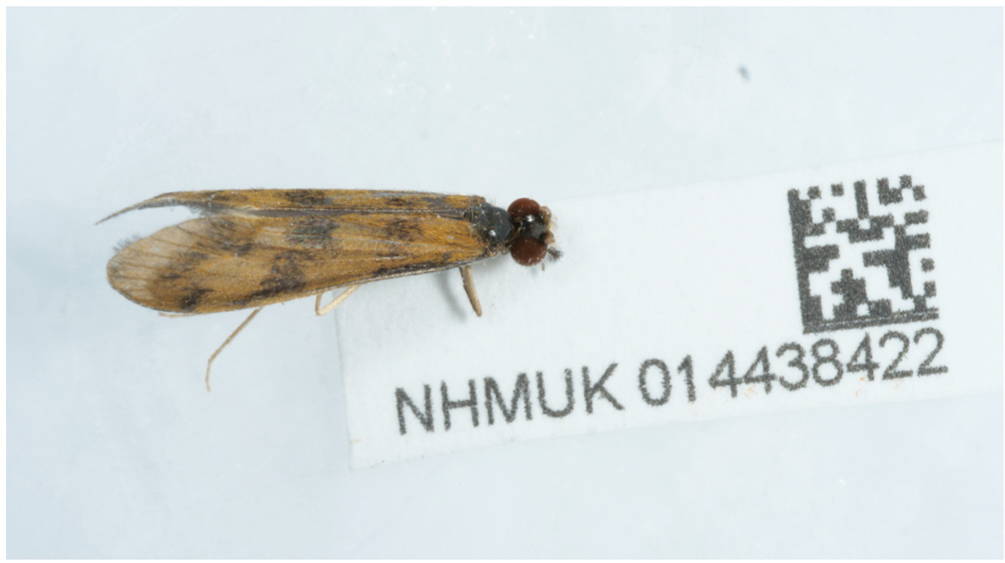
Photograph of the *Mystacides longicornis* (iiMysLong1) specimen used for genome sequencing.

The genome was sequenced from a male *Mystacides longicornis* ( Figure 1) collected from Lea Broad, England, UK (52.62, 1.23). A total of 43-fold coverage in Pacific Biosciences single-molecule HiFi long reads was generated. Primary assembly contigs were scaffolded with chromosome conformation Hi-C data. Manual assembly curation corrected 13 missing joins or mis-joins and removed 2 haplotypic duplications, reducing the scaffold number by 13.64%, and increasing the scaffold N50 by 0.34%.

**Figure 2.**
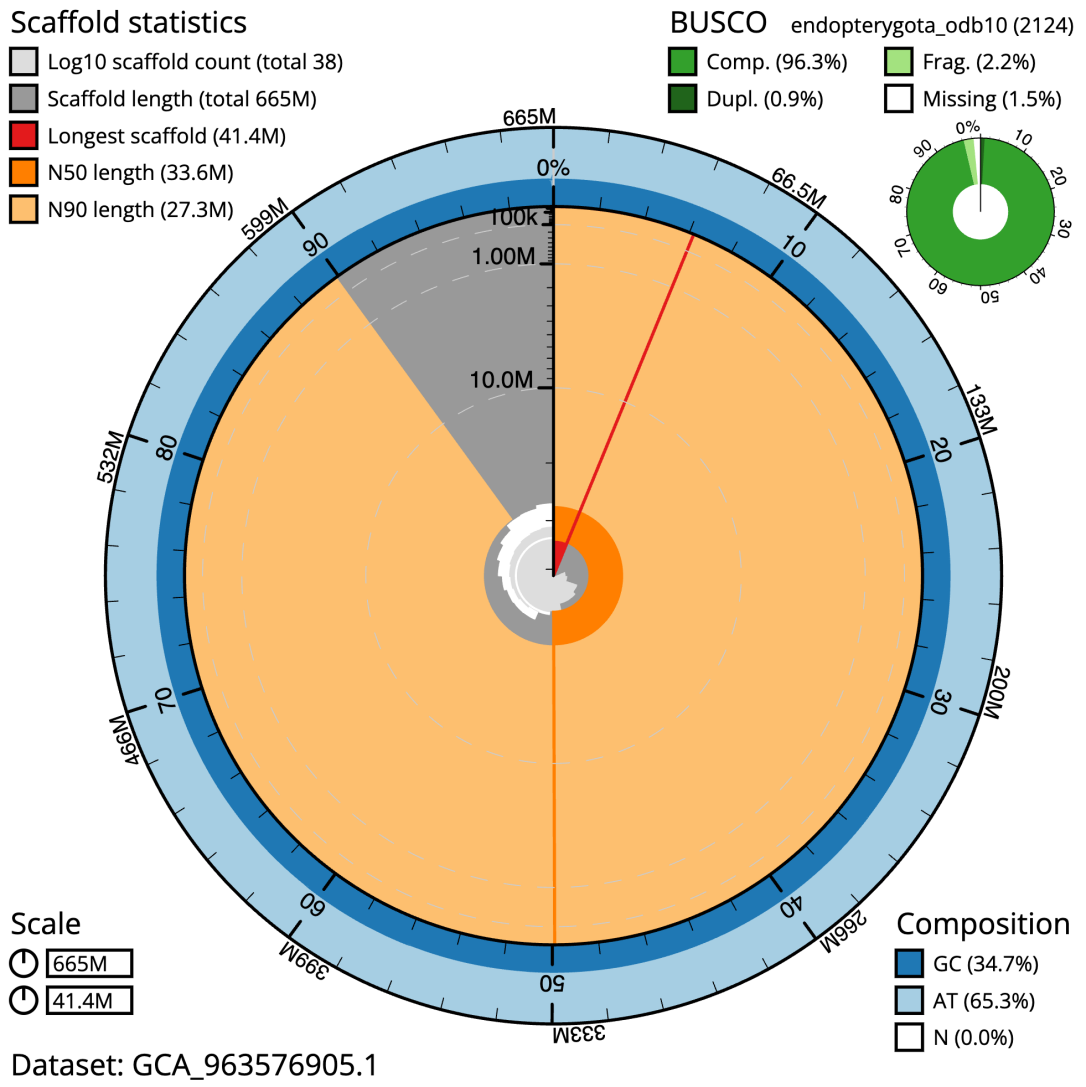
Genome assembly of *Mystacides longicornis*, iiMysLong1.1: metrics. The BlobToolKit snail plot shows N50 metrics and BUSCO gene completeness. The main plot is divided into 1,000 size-ordered bins around the circumference with each bin representing 0.1% of the 665,109,236 bp assembly. The distribution of sequence lengths is shown in dark grey with the plot radius scaled to the longest sequence present in the assembly (41,430,793 bp, shown in red). . Orange and pale-orange arcs show the N50 and N90 sequence lengths (33,624,892 and 27,270,352 bp), respectively. The pale grey spiral shows the cumulative sequence count on a log scale with white scale lines showing successive orders of magnitude. The blue and pale-blue area around the outside of the plot shows the distribution of GC, AT and N percentages in the same bins as the inner plot. A summary of complete, fragmented, duplicated and missing BUSCO genes in the endopterygota_odb10 set is shown in the top right. An interactive version of this figure is available at https://blobtoolkit.genomehubs.org/view/Mystacides_longicornis/dataset/GCA_963576905.1/snail.

**Figure 3.**
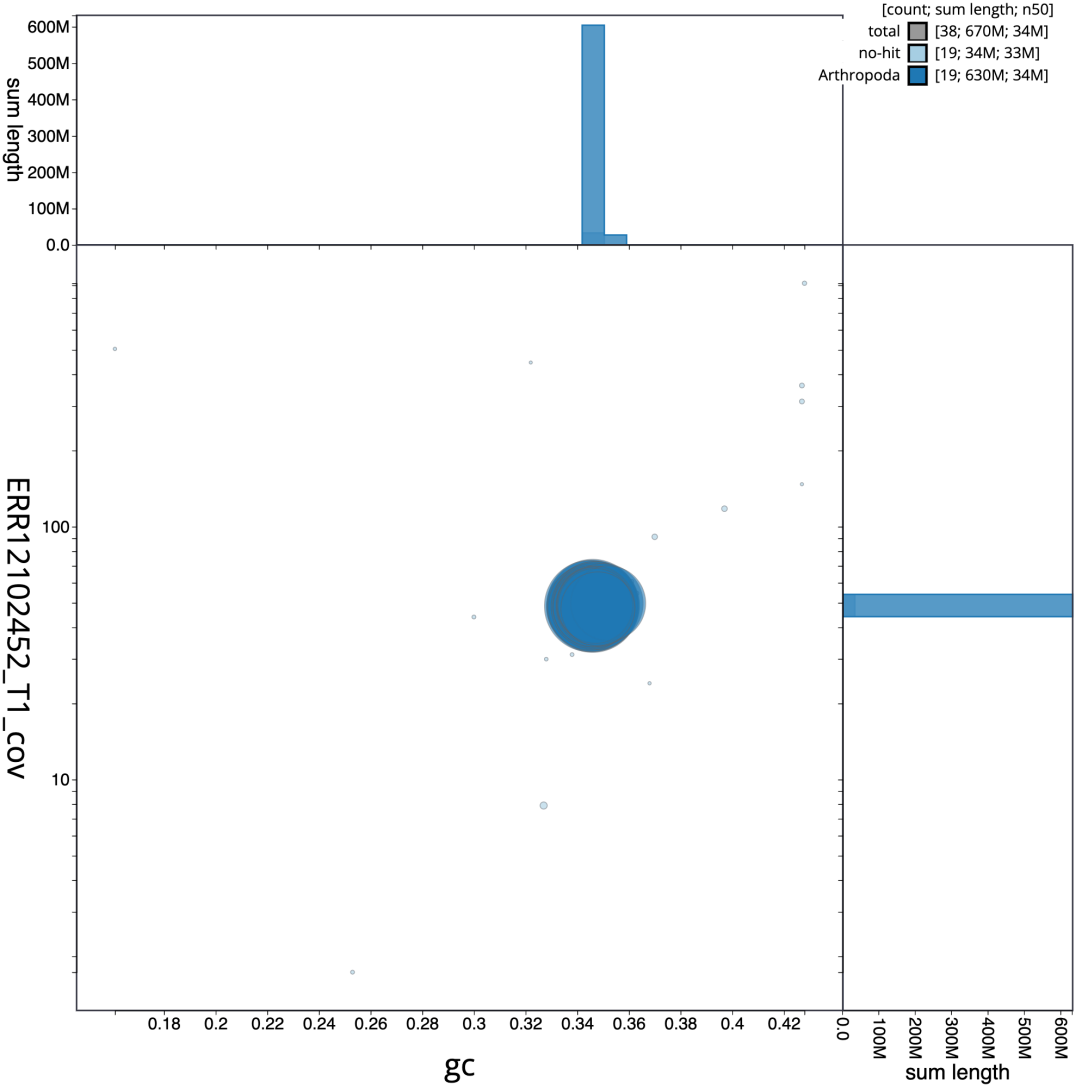
Genome assembly of *Mystacides longicornis*, iiMysLong1.1: BlobToolKit GC-coverage plot. Sequences are coloured by phylum. Circles are sized in proportion to sequence length. Histograms show the distribution of sequence length sum along each axis. An interactive version of this figure is available at https://blobtoolkit.genomehubs.org/view/Mystacides_longicornis/dataset/GCA_963576905.1/blob.

**Figure 4.**
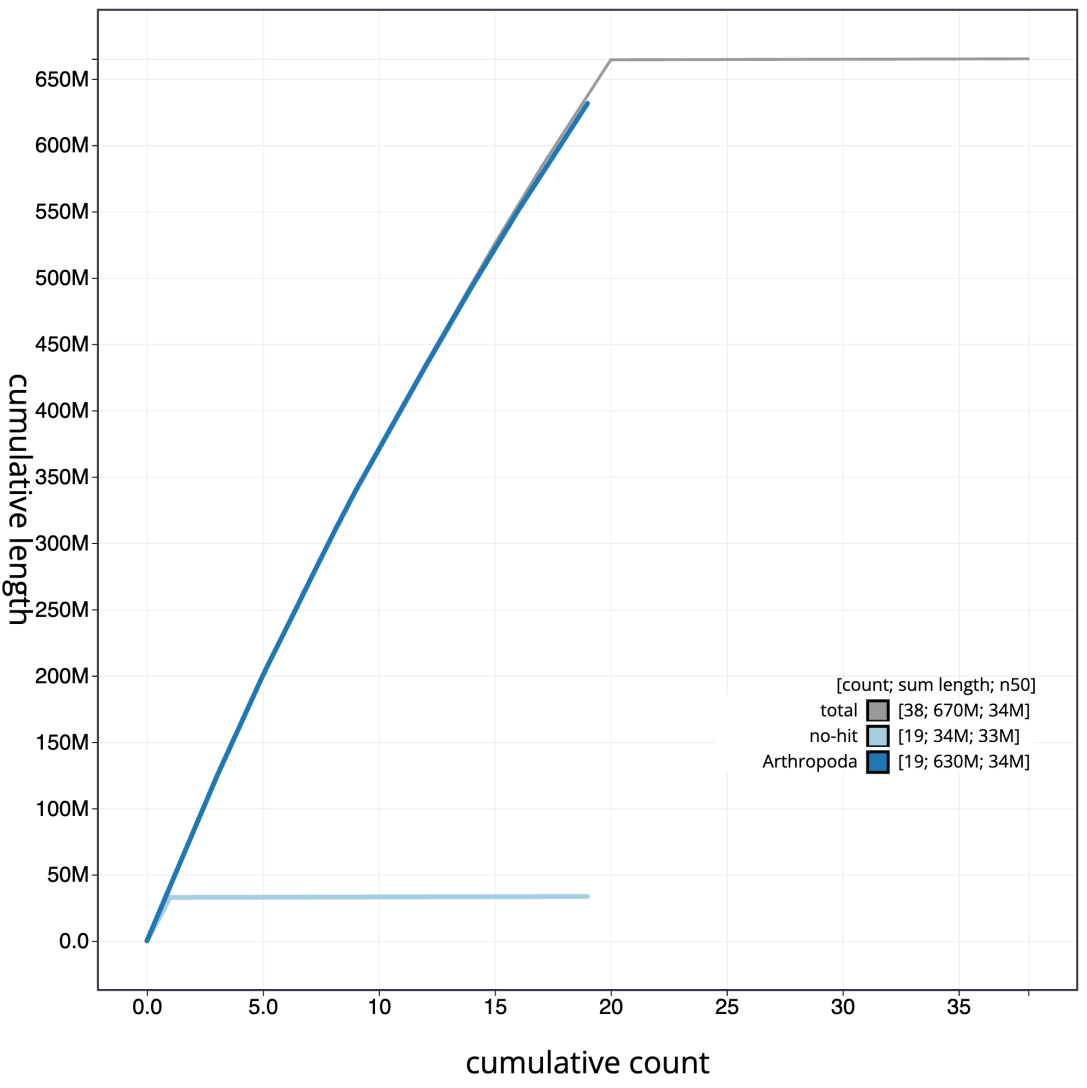
Genome assembly of *Mystacides longicornis*, iiMysLong1.1: BlobToolKit cumulative sequence plot. The grey line shows cumulative length for all sequences. Coloured lines show cumulative lengths of sequences assigned to each phylum using the buscogenes taxrule. An interactive version of this figure is available at https://blobtoolkit.genomehubs.org/view/Mystacides_longicornis/dataset/GCA_963576905.1/cumulative.

**Figure 5.**
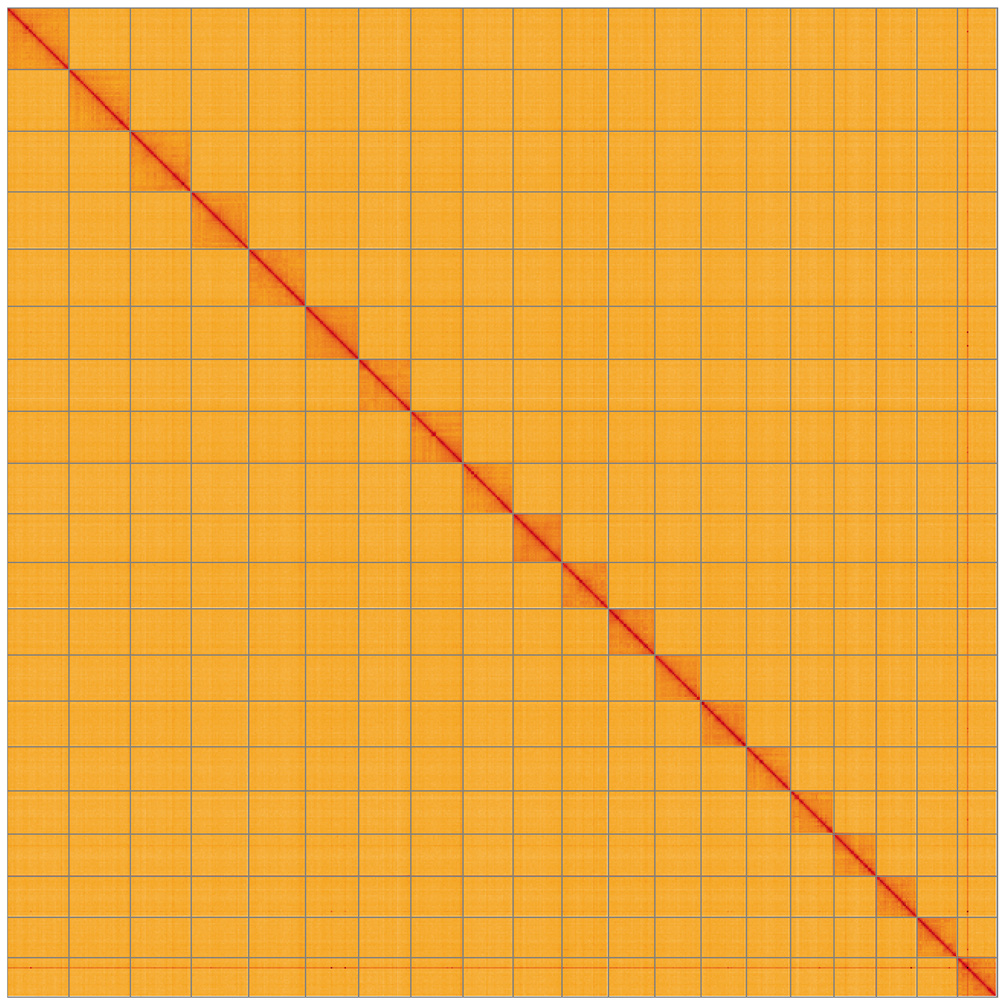
Genome assembly of *Mystacides longicornis*, iiMysLong1.1: Hi-C contact map of the iiMysLong1.1 assembly, visualised using HiGlass. Chromosomes are shown in order of size from left to right and top to bottom. An interactive version of this figure may be viewed at https://genome-note-higlass.tol.sanger.ac.uk/l/?d=Ndau4nLsQF2mSl7UaJ5hCA.

The final assembly has a total length of 665.1 Mb in 37 sequence scaffolds with a scaffold N50 of 33.6 Mb ( Table 1). The snail plot in Figure 2 provides a summary of the assembly statistics, while the distribution of assembly scaffolds on GC proportion and coverage is shown in Figure 3. The cumulative assembly plot in Figure 4 shows curves for subsets of scaffolds assigned to different phyla. Most (99.88%) of the assembly sequence was assigned to 20 chromosomal-level scaffolds, representing 19 autosomes and the Z sex chromosome. Chromosome-scale scaffolds confirmed by the Hi-C data are named in order of size ( Figure 5; Table 2). Chromosome Z was assigned based on synteny to *Athripsodes cinereus* (GCA_947579605.1) ( Wallace *et al.*, 2023). While not fully phased, the assembly deposited is of one haplotype. Contigs corresponding to the second haplotype have also been deposited. The mitochondrial genome was also assembled and can be found as a contig within the multifasta file of the genome submission.

**Table 1.** Genome data for *Mystacides longicornis*, iiMysLong1.1.

Project accession data		
Assembly identifier	iiMysLong1.1	
Species	*Mystacides longicornis*	
Specimen	iiMysLong1	
NCBI taxonomy ID	699848	
BioProject	PRJEB66746	
BioSample ID	SAMEA112964140	
Isolate information	iiMysLong1, whole organism (PacBio DNA and Illumina Hi-C sequencing)	
Assembly metrics *		*Benchmark*
Consensus quality (QV)	67.1	*≥ 50*
*k*-mer completeness	100.0%	*≥ 95%*
BUSCO **	C:96.0%[S:95.1%,D:0.9%], F:2.4%,M:1.6%,n:2,124	*C ≥ 95%*
Percentage of assembly mapped to chromosomes	99.88%	*≥ 95%*
Sex chromosomes	Z	*localised homologous pairs*
Organelles	Mitochondrial genome: 15.75 kb	*complete single alleles*
Raw data accessions		
PacificBiosciences Sequel IIe	ERR12102452	
Hi-C Illumina	ERR12102418	
Genome assembly		
Assembly accession	GCA_963576905.1	
*Accession of alternate haplotype*	GCA_963576805.1	
Span (Mb)	665.1	
Number of contigs	246	
Contig N50 length (Mb)	5.1	
Number of scaffolds	37	
Scaffold N50 length (Mb)	33.6	
Longest scaffold (Mb)	41.43	

* Assembly metric benchmarks are adapted from column VGP-2020 of “Table 1: Proposed standards and metrics for defining genome assembly quality” from Rhie *et al.* (2021). ** BUSCO scores based on the endopterygota_odb10 BUSCO set using version v5.4.3. C = complete [S = single copy, D = duplicated], F = fragmented, M = missing, n = number of orthologues in comparison. A full set of BUSCO scores is available at https://blobtoolkit.genomehubs.org/view/Mystacides_longicornis/dataset/GCA_963576905.1/busco.

**Table 2.** Chromosomal pseudomolecules in the genome assembly of Mystacides longicornis, iiMysLong1.

INSDC accession	Chromosome	Length (Mb)	GC%
OY756420.1	1	41.43	34.5
OY756421.1	2	41.25	34.5
OY756422.1	3	40.82	34.5
OY756423.1	4	38.54	34.5
OY756424.1	5	38.16	34.5
OY756425.1	6	35.65	34.5
OY756426.1	7	35.03	34.5
OY756427.1	8	35.01	34.5
OY756428.1	9	33.62	34.5
OY756430.1	10	31.19	35.0
OY756431.1	11	31.14	34.5
OY756432.1	12	31.01	34.5
OY756433.1	13	30.46	34.5
OY756434.1	14	29.69	34.5
OY756435.1	15	29.09	34.5
OY756436.1	16	28.42	34.5
OY756437.1	17	27.27	35.0
OY756438.1	18	27.17	34.5
OY756439.1	19	26.59	35.0
OY756429.1	Z	32.82	34.5
OY756440.1	MT	0.02	16.0

The estimated Quality Value (QV) of the final assembly is 67.1 with *k*-mer completeness of 100.0%, and the assembly has a BUSCO v5.4.3 completeness of 96.0% (single = 95.1%, duplicated = 0.9%), using the endopterygota_odb10 reference set ( *n* = 2,124).

Metadata for specimens, BOLD barcode results, spectra estimates, sequencing runs, contaminants and pre-curation assembly statistics are given at https://links.tol.sanger.ac.uk/species/699848.

## Methods

### Sample acquisition and nucleic acid extraction

A male *Mystacides longicornis* (specimen ID NHMUK014438422, ToLID iiMysLong1) was hand-picked from Lea Broad, England, UK (latitude 52.62, longitude 1.23) on 2022-04-07. The specimen was hand-picked by Derek Coleman (Dipterists Forum), who also formally identified the species. It was preserved by dry-freezing at –80°C.

The workflow for high molecular weight (HMW) DNA extraction at the Wellcome Sanger Institute (WSI) Tree of Life Core Laboratory includes a sequence of core procedures: sample preparation; sample homogenisation, DNA extraction, fragmentation, and clean-up. The sample was prepared for DNA extraction at the WSI Tree of Life Core Laboratory: the iiMysLong1 sample was weighed and dissected on dry ice ( Jay *et al.*, 2023). Tissue of the whole organism was homogenised using a PowerMasher II tissue disruptor ( Denton *et al.*, 2023a), setting aside tissue for Hi-C sequencing.

HMW DNA was extracted in the WSI Scientific Operations core using the Automated MagAttract v2 protocol ( Oatley *et al.*, 2023). The DNA was sheared into an average fragment size of 12–20 kb in a Megaruptor 3 system with speed setting 31 ( Bates *et al.*, 2023). Sheared DNA was purified by solid-phase reversible immobilisation ( Strickland *et al.*, 2023): in brief, the method employs a 1.8X ratio of AMPure PB beads to sample to eliminate shorter fragments and concentrate the DNA. The concentration of the sheared and purified DNA was assessed using a Nanodrop spectrophotometer and Qubit Fluorometer and Qubit dsDNA High Sensitivity Assay kit. Fragment size distribution was evaluated by running the sample on the FemtoPulse system.

Protocols developed by the WSI Tree of Life laboratory are publicly available on protocols.io ( Denton *et al.*, 2023b).

### Sequencing

Pacific Biosciences HiFi circular consensus DNA sequencing libraries were constructed according to the manufacturers’ instructions. DNA sequencing was performed by the Scientific Operations core at the WSI on a Pacific Biosciences Sequel IIe (HiFi) instrument. Hi-C data were also generated from remaining tissue of iiMysLong1 using the Arima2 kit and sequenced on the Illumina NovaSeq 6000 instrument.

### Genome assembly and curation

Assembly was carried out with Hifiasm ( Cheng *et al.*, 2021) and haplotypic duplication was identified and removed with purge_dups ( Guan *et al.*, 2020). The assembly was then scaffolded with Hi-C data ( Rao *et al.*, 2014) using YaHS ( Zhou *et al.*, 2023). The assembly was checked for contamination and corrected using the TreeVal pipeline ( Pointon *et al.*, 2023). Manual curation was performed using JBrowse2 ( Diesh *et al.*, 2023), HiGlass ( Kerpedjiev *et al.*, 2018) and PretextView ( Harry, 2022). The mitochondrial genome was assembled using MitoHiFi ( Uliano-Silva *et al.*, 2023), which runs MitoFinder ( Allio *et al.*, 2020) or MITOS ( Bernt *et al.*, 2013) and uses these annotations to select the final mitochondrial contig and to ensure the general quality of the sequence.

### Final assembly evaluation

The final assembly was post-processed and evaluated with the three Nextflow ( Di Tommaso *et al.*, 2017) DSL2 pipelines “sanger-tol/readmapping” ( Surana *et al.*, 2023a), “sanger-tol/genomenote” ( Surana *et al.*, 2023b), and “sanger-tol/blobtoolkit” ( Muffato *et al.*, 2024). The pipeline sanger-tol/readmapping aligns the Hi-C reads with bwa-mem2 ( Vasimuddin *et al.*, 2019) and combines the alignment files with SAMtools ( Danecek *et al.*, 2021). The sanger-tol/genomenote pipeline transforms the Hi-C alignments into a contact map with BEDTools ( Quinlan & Hall, 2010) and the Cooler tool suite ( Abdennur & Mirny, 2020), which is then visualised with HiGlass ( Kerpedjiev *et al.*, 2018). It also provides statistics about the assembly with the NCBI datasets ( Sayers *et al.*, 2024) report, computes *k*-mer completeness and QV consensus quality values with FastK and MerquryFK, and a completeness assessment with BUSCO ( Manni *et al.*, 2021).

The sanger-tol/blobtoolkit pipeline is a Nextflow port of the previous Snakemake Blobtoolkit pipeline ( Challis *et al.*, 2020). It aligns the PacBio reads with SAMtools and minimap2 ( Li, 2018) and generates coverage tracks for regions of fixed size. In parallel, it queries the GoaT database ( Challis *et al.*, 2023) to identify all matching BUSCO lineages to run BUSCO ( Manni *et al.*, 2021). For the three domain-level BUSCO lineage, the pipeline aligns the BUSCO genes to the Uniprot Reference Proteomes database ( Bateman *et al.*, 2023) with DIAMOND ( Buchfink *et al.*, 2021) blastp. The genome is also split into chunks according to the density of the BUSCO genes from the closest taxonomically lineage, and each chunk is aligned to the Uniprot Reference Proteomes database with DIAMOND blastx. Genome sequences that have no hit are then chunked with seqtk and aligned to the NT database with blastn ( Altschul *et al.*, 1990). All those outputs are combined with the blobtools suite into a blobdir for visualisation.

All three pipelines were developed using the nf-core tooling ( Ewels *et al.*, 2020), use MultiQC ( Ewels *et al.*, 2016), and make extensive use of the Conda package manager, the Bioconda initiative ( Grüning *et al.*, 2018), the Biocontainers infrastructure ( da Veiga Leprevost *et al.*, 2017), and the Docker ( Merkel, 2014) and Singularity ( Kurtzer *et al.*, 2017) containerisation solutions.

Table 3 contains a list of relevant software tool versions and sources.

**Table 3.** Software tools: versions and sources.

Software tool	Version	Source
BEDTools	2.30.0	https://github.com/arq5x/bedtools2
Blast	2.14.0	ftp://ftp.ncbi.nlm.nih.gov/blast/executables/blast+/
BlobToolKit	4.3.7	https://github.com/blobtoolkit/blobtoolkit
BUSCO	5.4.3 and 5.5.0	https://gitlab.com/ezlab/busco
bwa-mem2	2.2.1	https://github.com/bwa-mem2/bwa-mem2
Cooler	0.8.11	https://github.com/open2c/cooler
DIAMOND	2.1.8	https://github.com/bbuchfink/diamond
fasta_windows	0.2.4	https://github.com/tolkit/fasta_windows
FastK	427104ea91c78c3b8b8b49f1a7d6bbeaa869ba1c	https://github.com/thegenemyers/FASTK
GoaT CLI	0.2.5	https://github.com/genomehubs/goat-cli
Hifiasm	0.19.5-r587	https://github.com/chhylp123/hifiasm
HiGlass	44086069ee7d4d3f6f3f0012569789ec138f42b84a a44357826c0b6753eb28de	https://github.com/higlass/higlass
MerquryFK	d00d98157618f4e8d1a9190026b19b471055b22e	https://github.com/thegenemyers/MERQURY.FK
MitoHiFi	3	https://github.com/marcelauliano/MitoHiFi
MultiQC	1.14, 1.17, and 1.18	https://github.com/MultiQC/MultiQC
NCBI Datasets	15.12.0	https://github.com/ncbi/datasets
Nextflow	23.04.0-5857	https://github.com/nextflow-io/nextflow
PretextView	0.2	https://github.com/wtsi-hpag/PretextView
purge_dups	1.2.5	https://github.com/dfguan/purge_dups
samtools	1.16.1, 1.17, and 1.18	https://github.com/samtools/samtools
sanger-tol/genomenote	1.1.1	https://github.com/sanger-tol/genomenote
sanger-tol/readmapping	1.2.1	https://github.com/sanger-tol/readmapping
Seqtk	1.3	https://github.com/lh3/seqtk
Singularity	3.9.0	https://github.com/sylabs/singularity
TreeVal	1.0.0	https://github.com/sanger-tol/treeval
YaHS	1.2a.2	https://github.com/c-zhou/yahs

### Wellcome Sanger Institute – Legal and Governance

The materials that have contributed to this genome note have been supplied by a Darwin Tree of Life Partner. The submission of materials by a Darwin Tree of Life Partner is subject to the **‘Darwin Tree of Life Project Sampling Code of Practice’**, which can be found in full on the Darwin Tree of Life website here. By agreeing with and signing up to the Sampling Code of Practice, the Darwin Tree of Life Partner agrees they will meet the legal and ethical requirements and standards set out within this document in respect of all samples acquired for, and supplied to, the Darwin Tree of Life Project.

Further, the Wellcome Sanger Institute employs a process whereby due diligence is carried out proportionate to the nature of the materials themselves, and the circumstances under which they have been/are to be collected and provided for use. The purpose of this is to address and mitigate any potential legal and/or ethical implications of receipt and use of the materials as part of the research project, and to ensure that in doing so we align with best practice wherever possible. The overarching areas of consideration are:

•   Ethical review of provenance and sourcing of the material

•   Legality of collection, transfer and use (national and international)

Each transfer of samples is further undertaken according to a Research Collaboration Agreement or Material Transfer Agreement entered into by the Darwin Tree of Life Partner, Genome Research Limited (operating as the Wellcome Sanger Institute), and in some circumstances other Darwin Tree of Life collaborators.

## Data Availability

European Nucleotide Archive: *Mystacides longicornis*. Accession number PRJEB66746; https://identifiers.org/ena.embl/PRJEB66746 ( Wellcome Sanger Institute, 2023). The genome sequence is released openly for reuse. The *Mystacides longicornis* genome sequencing initiative is part of the Darwin Tree of Life (DToL) project. All raw sequence data and the assembly have been deposited in INSDC databases. The genome will be annotated using available RNA-Seq data and presented through the Ensembl pipeline at the European Bioinformatics Institute. Raw data and assembly accession identifiers are reported in Table 1.

## Author information

Members of the Natural History Museum Genome Acquisition Lab are listed here: https://doi.org/10.5281/zenodo.7139035.

Members of the Darwin Tree of Life Barcoding collective are listed here: https://doi.org/10.5281/zenodo.4893703.

Members of the Wellcome Sanger Institute Tree of Life Management, Samples and Laboratory team are listed here: https://doi.org/10.5281/zenodo.10066175.

Members of Wellcome Sanger Institute Scientific Operations: Sequencing Operations are listed here: https://doi.org/10.5281/zenodo.10043364.

Members of the Wellcome Sanger Institute Tree of Life Core Informatics team are listed here: https://doi.org/10.5281/zenodo.10066637.

Members of the Tree of Life Core Informatics collective are listed here: https://doi.org/10.5281/zenodo.5013541.

Members of the Darwin Tree of Life Consortium are listed here: https://doi.org/10.5281/zenodo.4783558.
