# Supplementary material for: The genome sequence of a cased caddisfly, Mystacides longicornis (Linnaeus, 1758)
Source: Wellcome Open Res. 2024 Jun 13;9:310. [Version 1] doi: 10.12688/wellcomeopenres.22272.1 (PMC11380072; doi:10.12688/wellcomeopenres.22272.1)
Supplement: Peer review history

# Reviewer report: Reviewer response for version 1

**DOI:** 10.21956/wellcomeopenres.24549.r94736

**Published:** 2024-09-20

**Authors:** Gurinder Kaur Walia

**Affiliations:** 1 Punjabi University, Patiala, Punjab, India

1. The genome of *Mystacides longicornis* should be compares to those of other related species. This can provide insights into evolutionary changes and genomic adaptations.
2. The article should also add the outlining of future research directions or potential applications of the genome data which provide a clearer picture of how the data can be utilized.

Are sufficient details of methods and materials provided to allow replication by others?

Yes

Is the rationale for creating the dataset(s) clearly described?

Yes

Are the datasets clearly presented in a useable and accessible format?

Yes

Are the protocols appropriate and is the work technically sound?

Yes

Reviewer Expertise:

molecular cytogenetics

I confirm that I have read this submission and believe that I have an appropriate level of expertise to confirm that it is of an acceptable scientific standard.

# Reviewer report: Reviewer response for version 1

**DOI:** 10.21956/wellcomeopenres.24549.r94734

**Published:** 2024-09-06

**Authors:** Doga Cedden

**Affiliations:** 1 University of Göttingen, Göttingen, Germany

The data note by Derek Coleman presents a genome assembly from *Mystacides longicornis *of sufficient quality.  The insect is adequately described, and the species taxonomy is accurate. The author presents a sufficient quality assessment of the assembly. The assembly was made publicly accessible. My only concern regarding the data note is that the antennae are not present in Figure 1 and should be one of the defining features of this species ( *longicornis*). Please consider replacing Figure 1. with a more appropriate photograph where the antennae are present and visible.

Are sufficient details of methods and materials provided to allow replication by others?

Yes

Is the rationale for creating the dataset(s) clearly described?

Yes

Are the datasets clearly presented in a useable and accessible format?

Yes

Are the protocols appropriate and is the work technically sound?

Yes

Reviewer Expertise:

I work on beetle pests using RNAi and transcriptomics.

I confirm that I have read this submission and believe that I have an appropriate level of expertise to confirm that it is of an acceptable scientific standard.

# Reviewer report: Reviewer response for version 1

**DOI:** 10.21956/wellcomeopenres.24549.r94741

**Published:** 2024-08-28

**Authors:** Ruiqi Li

**Affiliations:** 1 University of Colorado Boulder, Boulder, Colorado, USA

Coleman et al. have presented a high-quality genome of the cased caddisfly, *Mystacides longicornis* (Linnaeus, 1758). The study is well-conducted, and I have only a few minor suggestions for improvement.

 1. Introduction: It would be beneficial to include a short paragraph on the potential applications of the genome. For example, are there any notable biological features within the clade whose genetic mechanisms are not yet understood? Additionally, how might this genome contribute to species delimitation and systematics within the group?

 2. Figure 1: Adding a scale bar to Figure 1

 3. Sample Acquisition: In the "Sample acquisition" section, I suggest combining the two sentences mentioning that the specimen “… was hand-picked from Lea Broad by Derek Coleman…” to avoid potential confusion.

 3. Could you provide details on how the sample was prepared? Was the specimen snap-frozen, and if so, how was this process handled?

 4. RNA Sequencing: If RNA was sequenced as part of this study, please include details on how it was extracted.

 4. Did the authors verify the chromosome numbers? There are two other genomes available for Leptocerinae, both of which report 25 chromosomes (Wallace et al. 2023, Coleman et al. 2024). Typically, chromosome numbers are consistent among closely related species. For example, within the subfamily Tridacninae, most species have 18 chromosomes, with Tridacna gigas being a slight exception with 19 chromosomes, which is still very close to 18 (Li et al., 2024 ^3,4,5,6^). It would be helpful to double check the numbers.

Are sufficient details of methods and materials provided to allow replication by others?

Yes

Is the rationale for creating the dataset(s) clearly described?

Yes

Are the datasets clearly presented in a useable and accessible format?

Yes

Are the protocols appropriate and is the work technically sound?

Yes

Reviewer Expertise:

Genomics

I confirm that I have read this submission and believe that I have an appropriate level of expertise to confirm that it is of an acceptable scientific standard.

## References

[1] : The genome sequence of a cased caddisfly, Ceraclea dissimilis (Stephens, 1836). *Wellcome Open Research* .2024;9: 10.12688/wellcomeopenres.22268.1 10.12688/wellcomeopenres.22268.1 PMC1263552241278532

[2] : The genome sequence of the Brown Silverhorn, Athripsodes cinereus (Curtis, 1834). *Wellcome Open Research* .2023;8: 10.12688/wellcomeopenres.19765.1 10.12688/wellcomeopenres.19765.1 PMC1154419739525703

[3] : The genome sequence of the giant clam, Tridacna gigas (Linnaeus, 1758). *Wellcome Open Res* .2024;9: 10.12688/wellcomeopenres.21136.1 145 10.12688/wellcomeopenres.21136.1 38800516 PMC11116938

[4] : The genome sequence of the giant clam, Tridacna crocea (Lamarck, 1819). *Wellcome Open Research* .2023;8: 10.12688/wellcomeopenres.20580.1 10.12688/wellcomeopenres.20580.1

[5] : The genome sequence of the smooth giant clam, Tridacna derasa Röding, 1798. *Wellcome Open Research* .2024;9: 10.12688/wellcomeopenres.22618.1 10.12688/wellcomeopenres.22618.1 PMC1111693838800516

[6] : The genome sequence of the horse’s hoof clam, Hippopus hippopus (Linnaeus, 1758). *Wellcome Open Research* .2024;9: 10.12688/wellcomeopenres.21137.1 10.12688/wellcomeopenres.21137.1
